# A model to predict the risk of lethal nasopharyngeal necrosis after re-irradiation with intensity-modulated radiotherapy in nasopharyngeal carcinoma patients

**DOI:** 10.1186/s40880-016-0124-0

**Published:** 2016-06-29

**Authors:** Ya-Hui Yu, Wei-Xiong Xia, Jun-Li Shi, Wen-Juan Ma, Yong Li, Yan-Fang Ye, Hu Liang, Liang-Ru Ke, Xing Lv, Jing Yang, Yan-Qun Xiang, Xiang Guo

**Affiliations:** State Key Laboratory of Oncology in South China, Collaborative Innovation Center of Cancer Medicine, Sun Yat-sen University Cancer Center, Guangzhou, 510060 Guangdong P. R. China; Department of Nasopharyngeal Carcinoma, Sun Yat-sen University Cancer Center, Guangzhou, 510060 Guangdong P. R. China; Department of Biodevices and Diagnostics, Institute of Bioengineering and Nanotechnology, Singapore, 138669 Singapore; Department of Radiation Therapy, 3rd Affiliated Hospital of Guangzhou Medical University, Guangzhou, 510150 Guangdong P. R. China; Department of Pathology, Sun Yat-sen University Cancer Center, Guangzhou, 510060 Guangdong P. R. China; Department of Biostatistics and Epidemiology, School of Public Health, Sun Yat-sen University, Guangzhou, 510080 Guangdong P. R. China

**Keywords:** Nasopharyngeal carcinoma, Re-irradiation, Intensity-modulated radiotherapy, Necrosis

## Abstract

**Background:**

For patients with nasopharyngeal carcinoma (NPC) who undergo re-irradiation with intensity-modulated radiotherapy (IMRT), lethal nasopharyngeal necrosis (LNN) is a severe late adverse event. The purpose of this study was to identify risk factors for LNN and develop a model to predict LNN after radical re-irradiation with IMRT in patients with recurrent NPC.

**Methods:**

Patients who underwent radical re-irradiation with IMRT for locally recurrent NPC between March 2001 and December 2011 and who had no evidence of distant metastasis were included in this study. Clinical characteristics, including recurrent carcinoma conditions and dosimetric features, were evaluated as candidate risk factors for LNN. Logistic regression analysis was used to identify independent risk factors and construct the predictive scoring model.

**Results:**

Among 228 patients enrolled in this study, 204 were at risk of developing LNN based on risk analysis. Of the 204 patients treated, 31 (15.2%) developed LNN. Logistic regression analysis showed that female sex (*P* = 0.008), necrosis before re-irradiation (*P* = 0.008), accumulated total prescription dose to the gross tumor volume (GTV) ≥145.5 Gy (*P* = 0.043), and recurrent tumor volume ≥25.38 cm^3^ (*P* = 0.009) were independent risk factors for LNN. A model to predict LNN was then constructed that included these four independent risk factors.

**Conclusions:**

A model that includes sex, necrosis before re-irradiation, accumulated total prescription dose to GTV, and recurrent tumor volume can effectively predict the risk of developing LNN in NPC patients who undergo radical re-irradiation with IMRT.

## Background

In South China, especially in Guangdong Province, nasopharyngeal carcinoma (NPC) is a common head and neck cancer [1, 2]. It is a highly radiosensitive malignancy with a local recurrence rate of 10%–36% [3–5]. In the past several decades, various treatment modalities, including external-beam radiotherapy (EBRT), intracavitary brachytherapy, interstitial radioactive implantation, stereotactic radiosurgery, nasopharyngectomy, chemotherapy, and a combination of these methods, have been used to treat the patients with NPC. Each method has its optimal treatment population [6]; for patients with locally recurrent NPC, EBRT remains the most effective modality [7].

Intensity-modulated radiotherapy (IMRT) is considered a better technique for the treatment of recurrent NPC because of its lower normal tissue doses and more homogeneous target doses compared with conformal radiotherapy [8]. Currently, radical IMRT is the most effective treatment for patients with locally recurrent NPC. However, IMRT does carry a risk of late toxicities that should not be ignored. For patients re-irradiated with IMRT for recurrent NPC, the patients with irreversible nasopharyngeal necrosis have the largest proportion of reported severe late adverse events [9], which may result in massive nasopharyngeal hemorrhage and death. Therefore, for patients with recurrent NPC who have undergone re-irradiation, it is urgent to predict and avoid lethal nasopharyngeal necrosis (LNN). In this retrospective study, we examined the relationship between the clinical characteristics of re-irradiated NPC patients and the incidence of LNN, and we subsequently developed a predictive scoring model for LNN.

## Patients and methods

### Patient selection

Patients who underwent radical re-irradiation with IMRT for locally recurrent NPC between March 2001 and December 2011 and who had no evidence of distant metastasis were included in this study. All patients had weekly nasopharyngeal examinations with nasopharyngoscopy during IMRT treatment and were followed 1 month after IMRT, every 3 months for the first 2 years, every 6 months for 3 additional years, and annually thereafter. Each follow-up included a complete medical record review, physical examination, and nasopharyngoscopy examination. At least once a year, if necessary, patients underwent serum electrolyte and complete blood count tests as well as chest X-ray, ultrasound/computed tomography (CT) of the abdomen, and magnetic resonance imaging (MRI) of the head-and-neck region. This study was approved by the Ethics Committee of Sun Yat-sen University Cancer Center.

IMRT was conducted as previously reported [10, 11]. Patients were placed in a supine position on a custom-made head support and immobilized with a thermoplastic mask covering the head and upper neck. CT scanning from the superior border of the frontal sinus to 2 cm below the clavicular heads was performed with a 3-mm slice thickness. Imaging data were transferred to the Corvus inverse treatment planning system (NOMOS Corporation, Wexford, PA, USA). The gross tumor volume (GTV), clinical target volume (CTV), gross tumor volume of cervical lymph node (GTVnd), and organs at risk (OARs) were contoured slice by slice on CT images. The recurrent gross target of the nasopharynx and lymph nodes in the neck were determined according to CT/MRI and physical examinations. According to guidelines from the International Commission on Radiation Units and Measurements Report 62 (ICRU62) [12], CTV was defined as GTV plus a 0.5–1 cm margin; it also included the recurrent lymph nodes in the neck. OARs, including the brainstem, spinal cord, temporal lobes, lens, optic nerves, chiasm, parotid glands, temporomandibular joints, and mandible, were carefully contoured. Planning target volumes for GTV, GTVnd, and CTV were generated according to immobilization and localization uncertainties. According to our measurements, the immobilization uncertainty was 2 mm in the lateral direction and 1 mm in the anterior, posterior, superior, and inferior directions. Localization uncertainty was defined according to the involved organs’ mobility, which was 1–2 mm for both tumor targets and the surrounding normal tissues. The total uncertainty, as automatically generated by the Corvus system, was 1.4–2.8 mm in every orientation. All patients received full-course IMRT with 6-MV X-rays generated by a Clinac-600C linear accelerator (Varian Medical Systems, Palo Alto, CA, USA). As a general rule, the dose comprising 33% of contralateral OARs was limited to less than one-half of the tolerance dose, referring to a severe complication rate of 5% within 5 years of radiotherapy (TD5/5). The dose of the ipsilateral side was not constrained as that of the contralateral side. Dose verification was carried out before re-irradiation. The dose error between the measurement and the plan was less than 3%.

### Diagnosis of nasopharyngeal necrosis

Diagnosis of nasopharyngeal necrosis was based on the clinical characteristics, including foul nasal smell, refractory headache, filemot necrotic tissue and skull base osteoradionecrosis in nasopharyngeal cavity under endoscopy, discontinuous nasopharyngeal mucosa line and/or tissue defects on MRI, and a heap of red-stained substance without cellular structure in hematoxylin-eosin staining under pathologic examination [11, 13–19]. Patients who died from intractable epistaxis diagnosed with nasopharyngeal necrosis were recorded as having LNN.

### Study parameters

Potential clinical characteristics, including sex, age, body mass index (BMI) at recurrence, recurrent stage, interval of recurrence, concurrent chemotherapy during re-irradiation, necrosis before re-irradiation, initial total radiation dose and fractionated dose, re-irradiation prescription dose to the GTV, mean re-irradiation dose, re-irradiation fractionated dose, duration of re-irradiation, accumulated total prescription dose to the GTV (initial and recurrent), and recurrent tumor volume, were recorded. The recurrent stage was reclassified according to the 2009 American Joint Committee on Cancer staging system. The interval of recurrence was defined from the last day of initial radiotherapy to the first day of re-irradiation. Necrosis before re-irradiation was defined as necrosis detected before the first day of re-irradiation. Duration of re-irradiation was calculated from the first day to the last day of re-irradiation.

### Statistical methods

All statistical analyses were performed using SPSS 22.0 software (SPSS, Chicago, IL, USA). Receiver operating characteristic curves based on LNN were applied to determine the cutoff points for the continuous variables. Logistic regression analysis was performed to estimate odds ratios (ORs) or adjusted odds ratios (AORs), 95% Confidence Intervals (CIs) and *P* values. Univariate logistic regression analysis was performed to identify potential risk factors for LNN. Multivariate logistic regression analysis was performed to distinguish the independent risk factors for LNN from the variables with statistical significance in the univariate logistic regression analysis. Multivariate logistic regression analysis was also used to construct a predictive model for LNN. All statistical tests were two-sided. *P* values less than 0.05 were considered statistically significant.

## Results

### Clinical characteristics

**Fig. 1:**
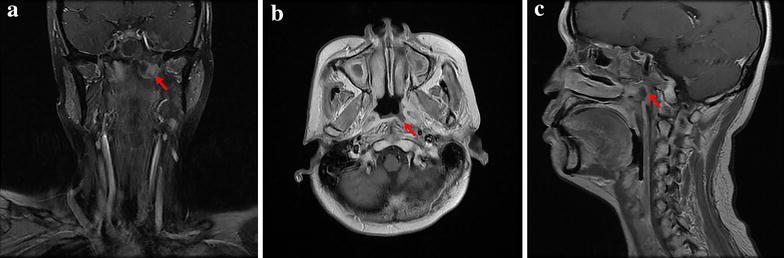
Magnetic resonance imaging of nasopharyngeal necrosis. **a** Coronal, contrast-enhanced, T1-weighted image shows non-enhanced soft tissues (like a hole) mixed with tiny air bubbles in the left nasopharyngeal lateral recess (*arrow*). **b** Transverse, contrast-enhanced, T1-weighted image shows the necrotic extent in the nasopharyngeal posterior wall (*arrow*). **c** Sagittal, contrast-enhanced, T1-weighted image shows an obvious defect in the left nasopharyngeal lateral recess (*arrow*)

**Fig. 2:**
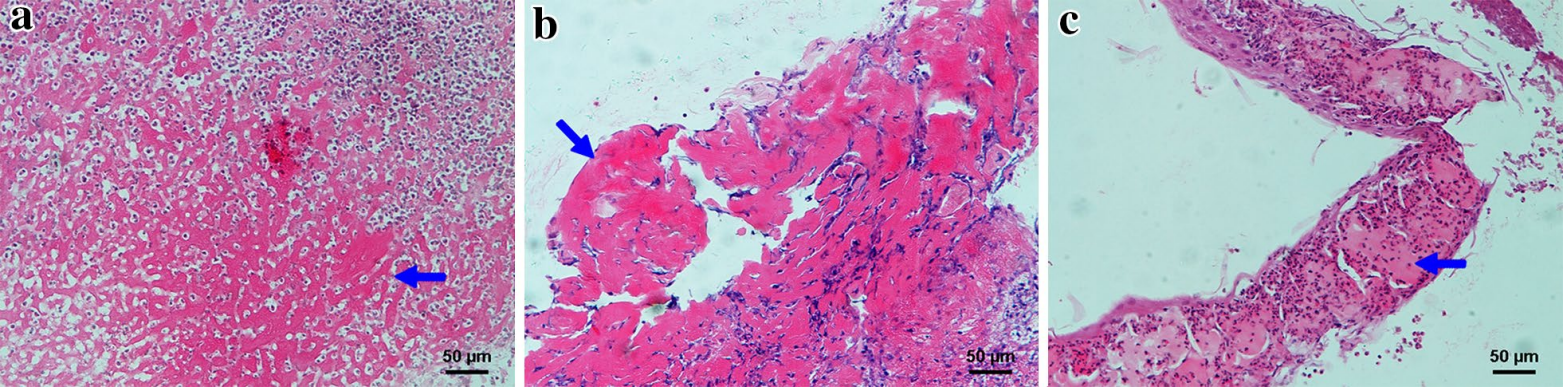
Pathologic characteristics of nasopharyngeal necrosis. The three necrotic tissues (**a**–**c**) were taken from three NPC patients. Hematoxylin–eosin staining of nasopharyngeal necrosis is similar that shows many *red-stained* substances without cellular structure (*arrows*)

**Fig. 3:**
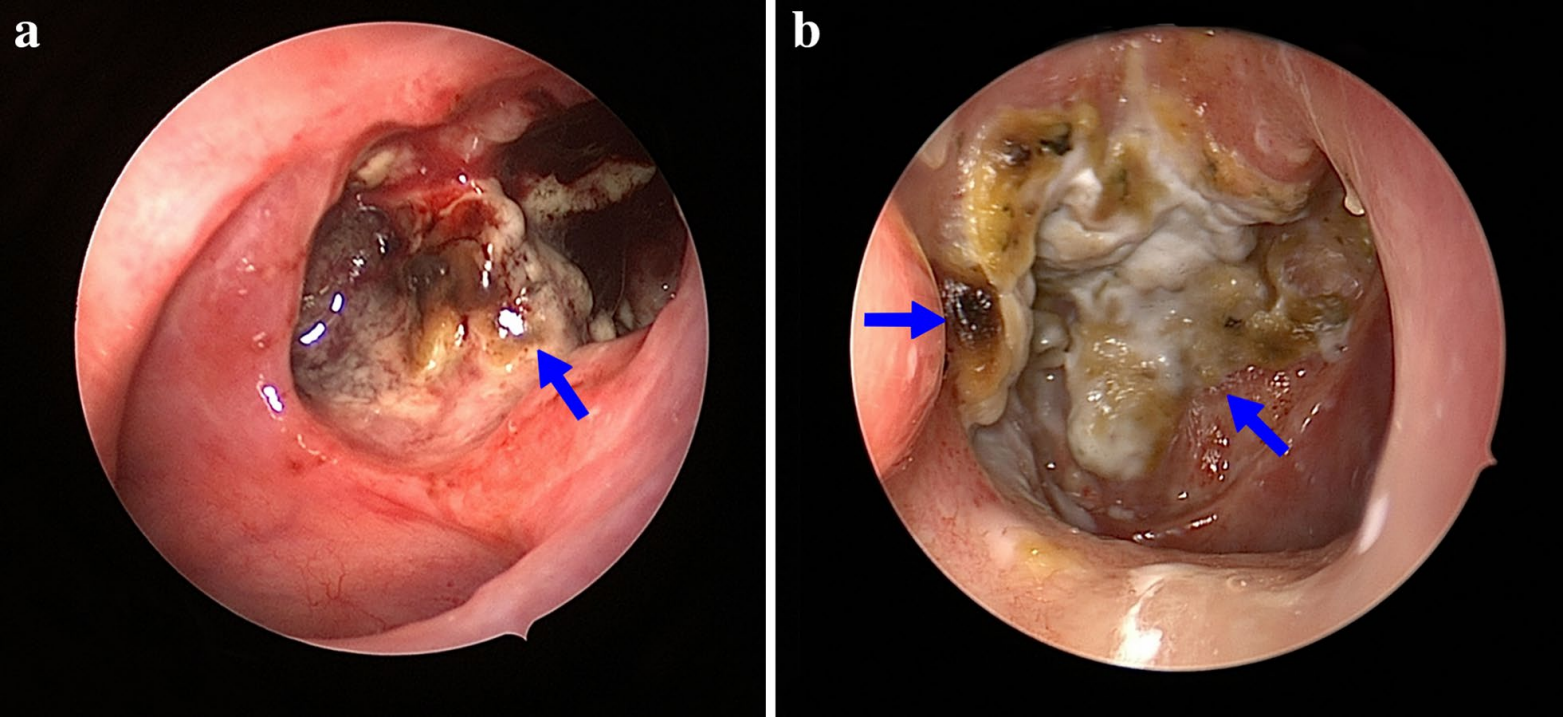
Endoscopic examination of nasopharyngeal necrosis. Nasopharyngeal necrosis is shown under endoscopic examination (*arrows*). **a** The necrosis is located in the roof, posterior, and left wall with obvious mucosa ulcer. Sequestra and necrotic bones can also be seen in the nasopharyngeal cavity. **b** The nasopharyngeal cavity is covered with *yellow* to *black* purulent secretion with necrosis located in the roof, posterior, and right lateral wall of the nasopharynx

In total, 228 patients were included in this study; among these, 24 patients were excluded from further analyses because the initial radiation dose was not available. Of the eligible 204 patients, 169 (82.8%) were diagnosed based on pathologic analysis with locally recurrent NPC; the remaining 35 (17.2%) were clinically diagnosed based on symptoms, MRI, or CT images. The median BMI of patients was 21.8 kg/m^2^ (range, 14.2–31.3 kg/m^2^), with a median age of 46 years (range, 21–79 years). All patients underwent conventional radiotherapy with 2 Gy/fraction per day, 5 days every week, in the initial treatment; the median isocenter dose to the nasopharynx was 70 Gy (range, 56–83 Gy). The median interval of recurrence was 30 months (range, 9–216 months). All patients completed the full-course radical IMRT of re-irradiation with the median re-irradiation prescription dose to the GTV of 64 Gy (range, 58–70 Gy), 2.14 Gy (range, 1.88–2.33 Gy)/fraction per day, 5 days every week. The duration of re-irradiation was 19–61 days (median, 43 days). The mean re-irradiation GTV dose was 61.63–77.44 Gy (median, 69.23 Gy), with a median recurrent volume of 43.81 cm^3^ (range, 2.98–197.99 cm^3^). During follow-up, 77 patients (37.7%) were diagnosed with nasopharyngeal necrosis; of these, 51 had died. Of those patients who had died, 31 (60.8%) died of intractable epistaxis, which was determined to be LNN; 6 (11.8%) died of distant metastasis; 11 (21.5%) died of local–regional recurrence; and 3 (5.9%) died of sequelae, such as difficulty in feeding, encephalatrophy, and other medical complications. Cutoff points of the continuous variables were analyzed based on LNN; the distribution of patients according to the cutoff points is shown in Table 1. The respective manifestations of nasopharyngeal necrosis on MRI, pathologic examination, and endoscopy are shown in Figs. 1, 2 and 3.

**Table 1:** Characteristics of 204 patients with locally recurrent nasopharyngeal carcinoma

Characteristic	All (*n* = 204)	LNN (*n* = 31)	Non-LNN (*n* = 173)
Sex			
Men	160 (78.4)	18 (58.1)	142 (82.1)
Women	44 (21.6)	13 (41.9)	31 (17.9)
Age (years)			
<39.5	48 (23.5)	4 (12.9)	44 (25.4)
≥39.5	156 (76.5)	27 (87.1)	129 (74.6)
Initial radiation dose (Gy)			
<74.68	178 (87.3)	25 (80.6)	153 (88.4)
≥74.68	26 (12.7)	6 (19.4)	20 (11.6)
Recurrent stage			
I	13 (6.4)	0 (0)	13 (7.5)
II	25 (12.3)	4 (12.9)	21 (12.1)
III	78 (38.2)	13 (41.9)	65 (37.6)
IV	88 (43.1)	14 (45.2)	74 (42.8)
Interval of recurrence (months)			
<25.5	73 (35.8)	9 (29.0)	64 (37.0)
≥25.5	131 (64.2)	22 (72.0)	109 (63.0)
CCRT during re-irradiation			
Yes	67 (32.8)	8 (25.8)	59 (34.1)
No	137 (67.2)	23 (74.2)	114 (65.9)
Necrosis before re-irradiation			
Yes	41 (20.1)	12 (38.7)	29 (16.8)
No	163 (79.9)	19 (61.3)	144 (83.2)
Re-irradiation dose (Gy)			
<67	135 (66.2)	18 (58.1)	117 (67.6)
≥67	69 (33.8)	13 (41.9)	56 (32.4)
Mean re-irradiation dose (Gy)			
<71.97	152 (74.5)	18 (58.1)	134 (77.5)
≥71.97	52 (25.5)	13 (41.9)	39 (22.5)
Re-irradiation fractionated dose (Gy)			
<2.3	197 (96.6)	30 (96.8)	167 (96.5)
≥2.3	7 (3.4)	1 (3.2)	6 (3.5)
Duration of re-irradiation (days)			
<49.5	173 (84.8)	23 (74.2)	150 (86.7)
≥49.5	31 (15.2)	8 (25.8)	23 (13.3)
Accumulated total GTV dose (Gy)			
<141.5	186 (91.2)	25 (80.6)	161 (93.1)
≥141.5	18 (8.8)	6 (19.4)	12 (6.9)
Recurrent tumor volume (cm^3^)			
<25.38	59 (28.9)	3 (9.7)	56 (32.4)
≥25.38	145 (71.1)	28 (90.3)	117 (67.6)
BMI			
<19.8	50 (24.5)	6 (19.4)	44 (25.4)
≥19.8	154 (75.5)	25 (80.6)	129 (74.6)
Necrosis			
Yes	77 (37.7)	31 (100)	46 (26.6)
No	127 (62.2)	0 (0)	127 (73.4)

All values are presented as the number of patients followed by percentage in the parentheses

*LNN* lethal nasopharyngeal necrosis, *CCRT* concurrent chemoradiotherapy, *GTV* gross tumor volume, *BMI* body mass index

### Univariate logistic regression analysis

Clinical characteristics, including sex, age, BMI at recurrence, recurrent stage, interval of recurrence, concurrent chemotherapy during re-irradiation, necrosis before re-irradiation, initial total radiation dose and fractionated dose, re-irradiation prescription dose to the GTV, mean re-irradiation dose, re-irradiation fractionated dose, duration of re-irradiation, accumulated total prescription dose to the GTV, and recurrent tumor volume, were subjected to univariate logistic regression analysis to determine their associations with LNN. Binary univariate regression analysis showed that female sex (OR 3.308; 95% CI 1.468–7.454; *P* = 0.004), necrosis before re-irradiation (OR 3.136; 95% CI 1.374–7.160; *P* = 0.007), mean re-irradiation dose ≥71.97 Gy (OR 2.481; 95% CI 1.118–5.509; *P* = 0.026), accumulated total GTV dose ≥145.50 Gy (OR 3.220; 95% CI 1.108–9.357; *P* = 0.032), and recurrent tumor volume ≥25.38 cm^3^ (OR 4.467; 95% CI 1.30–15.322; *P* = 0.017) were associated with LNN (Table 2).

**Table 2:** Univariate and multivariate regression analysis on the association between patient characteristics and LNN

Variable	Univariate analysis			Multivariate analysis		
	OR	95% CI	*P*	AOR	95% CI	*P*
Sex (women vs. men)	3.308	1.468–7.454	0.004	3.354	1.367–8.231	0.008
Age (≥39.5 vs. <39.5 years)	2.302	0.763–6.947	0.139	–	–	–
Initial radiation dose (≥74.68 vs. <74.68 Gy)	1.836	0.672–5.018	0.236	–	–	–
Recurrent stage (I vs. II vs. III vs. IV)	–	–	0.999	–	–	–
Interval of recurrence (≥25.5 vs. <25.5 months)	1.435	0.623–3.207	0.396	–	–	–
CCT during re-irradiation (yes vs. no)	0.672	0.283–1.594	0.367	–	–	–
Necrosis before re-irradiation (yes vs. no)	3.136	1.374–7.160	0.007	3.469	1.382–8.710	0.008
Re-irradiation GTV dose (≥ 67 vs. < 67 Gy)	1.509	0.691–3.296	0.302	–	–	–
Mean re-irradiation dose (≥71.97 vs. < 71.97 Gy)	2.481	1.118–5.509	0.026	1.713	0.691–4.248	0.246
Re-irradiation fractionated dose (≥ 2.3 vs. < 2.3 Gy)	–	–	0.999	–	–	–
Duration of re-irradiation (≥ 49.5 vs. < 49.5 days)	2.268	0.907–5.672	0.08	–	–	–
Accumulated total GTV dose (≥141.5 vs. <141.5 Gy)	3.220	1.108–9.357	0.032	3.763	1.041–13.611	0.043
Recurrent tumor volume (≥25.38 vs. <25.38 cm^3^)	4.467	1.302–15.322	0.017	6.063	1.561–23.543	0.009
BMI (≥19.8 vs. <19.8)	1.421	0.547–3.691	0.470	–	–	–

*LNN* lethal nasopharyngeal necrosis, *CI*, confidence interval, *CCT* concurrent chemoradiotherapy, *GTV* gross tumor volume, *BMI* body mass index, *OR* odds ratio, *AOR* adjusted odds ratio

### Multivariate regression analysis and the predictive model

To determine the independent risk factors for LNN, the five factors that were statistically significant were included in the multivariate regression analysis model with Enter method. Sex (AOR 3.354; 95% CI 1.367–8.231; *P* = 0.008), necrosis before re-irradiation (AOR 3.469; 95% CI 1.382–8.710; *P* = 0.008), accumulated total GTV dose (AOR 3.763; 95% CI 1.041–13.611; *P* = 0.043), and recurrent tumor volume (AOR 6.063; 95% CI 1.561–23.543; *P* = 0.009) were the independent risk factors for LNN (Table 2). The corresponding regression equation was as below:

$$ {\text{Logit}}\pi = 1. 2 2 9 {\text{X}}_{ 1} + 1. 2 7 6 {\text{ X}}_{ 2} + 1. 5 4 8 {\text{X}}_{ 3} + 1. 8 2 7 {\text{X}}_{ 4} - 4.0 7 2 $$

Note: X_1_ = sex (men = 0; women = 1); X_2_ = necrosis before re-irradiation (no = 0; yes = 1); X_3_ = total GTV dose (<141.50 Gy = 0; ≥141.50 Gy = 1); and X_4_ = recurrent volume (<25.38 cm^3^ = 0; ≥25.38 cm^3^ = 1). Logitπ = ln (π/1 − π); π means the risk of developing LNN of corresponding logit value.

The logitπ value for each patient was calculated according to the scoring model and graded quarterly by the corresponding estimated risk of developing LNN (Table 3). The probability of patients with logitπ ≥1.099 developing LNN was over 75% after re-irradiation with radical IMRT. However, the probability of developing LNN was less than 25% when logitπ <−1.099. In this study, 12 patients were assigned to the high-risk group which was defined as risk degree IV, and seven patients actually developed LNN (Table 3).

**Table 3:** The risk of developing LNN according to the model

Risk degree	No. of total patients	Risk of developing LNN	Value of logitπ	No. of LNN patients
I	56	<25%	<−1.099	1
II	90	25%–50%	−1.099 to 0	11
III	46	50%–75%	0–1.099	12
IV	12	≥75%	≥1.099	7

Logitπ was calculated from the model and classified into four degrees according to the corresponding risk of developing LNN

*LNN* lethal nasopharyngeal necrosis

## Discussion

In the present study, for patients with NPC who were treated with radical IMRT, we found that sex, necrosis before re-irradiation, accumulated total prescription dose to the GTV, and recurrent tumor volume are risk factors for LNN. We therefore constructed a feasible prognostic model that included these factors to predict the risk of developing LNN.

IMRT has been proven to improve tumor control and decrease acute and late toxicities when compared with conventional radiation technology [7, 8, 10, 20–22]. However, some severe late toxicities of IMRT, especially LNN, have adversely affected patients’ quality of life and survival. According to previous studies, 11%–32% of NPC patients with severe late adverse events underwent re-irradiation with IMRT, which resulted in nasopharyngeal necrosis [9, 15, 21]. Even worse, approximately 45% of nasopharyngeal necrosis later involved the internal carotid artery and resulted in LNN [22]. Hemostasis by gelatin sponge compression and nasopharyngeal packing through the anterior and/or posterior nares are the standard therapies. Internal/external carotid artery ligation, stent implantation, and other surgery treatments are applied when necessary [23–27]. Unfortunately, the cure rate remains very low despite the hemostasis. It is urgent to identify re-irradiated NPC patients who are at high risk of developing LNN.

Many studies cited dosage as an important risk factor for the severity of necrosis [13–15, 17, 19, 23]. Bedwinek et al. [19] reported that osteonecrosis occurred in 9% of patients with oral carcinoma and NPC who received radiotherapy if the dose was more than 70 Gy. Similarly, Mark et al. [17] observed that 22% of patients who received a dose greater than 75 Gy experienced osteoradionecrosis. In their study, 28 patients (18.4%) experienced nasopharyngeal necrosis after initial irradiation with a dose over 70 Gy. However, the incidence was much higher for re-irradiated patients. Hua et al. [15] reported that 14 of 28 (50%) patients developed nasopharyngeal necrosis after re-irradiation with an accumulated prescription dose over 120 Gy. In our study, univariate analysis showed that both the accumulated prescription dose to the GTV and the mean re-irradiation dose were statistically related to LNN, and multivariate analysis showed that the accumulated prescription dose to the GTV was an independent risk factor for LNN. Additionally, large recurrent tumor volume is usually considered one of the independent factors of poor survival in recurrent NPC [28]. Han et al. [10] showed that the OS of NPC patients with small recurrent tumor volume (≤38 cm^3^) was 1.6 times longer than that of patients with larger recurrent tumor volume (>38 cm^3^) when treated with IMRT. Moreover, Hua et al. [11] found that the recurrent tumor volume >42 cm^3^ was an independent predictor of OS in patients with locally recurrent NPC who were re-irradiated with IMRT. In our study, we found that a recurrent tumor volume ≥25.38 cm^3^ was an independent prognostic factor for LNN; this tumor volume was much lower than those in previous reports discussing survival status, suggesting that re-irradiation of large recurrent tumors should be administered with caution. Re-irradiation is usually the last chance for cure after a patient’s first relapse; therefore, for patients at high risk of developing LNN, decreasing the re-irradiation dose or the recurrent tumor volume might be feasible methods to prevent LNN. Considering that reducing the re-irradiation dose would likely result in worse control of NPC recurrence, administering neoadjuvant chemotherapy to decrease the tumor volume may lower the risk of developing LNN and improve the local control rate and the survival rate [29, 30]. Future clinical trials combining chemotherapy, molecular targeted therapy, and radiotherapy are expected to determine the optimal re-irradiation dose for patients with recurrent NPC.

A generally accepted mechanism of injury after irradiation is a hypovascular–hypoxic–hypocellular condition that causes the breakdown of local tissue, exposing bone, and the formation of sequestra [31]. The inflammation caused by this non-healing wound may, in turn, increase the demand of the local tissue for energy, oxygen, and other metabolites, which may lead to more serious collagen destruction and cell death [17]. This is a reasonable explanation for our findings. We found that necrosis before re-irradiation was an independent risk factor for LNN. Of the 41 patients with nasopharyngeal necrosis after the first irradiation, 12 (29.3%) progressed to LNN after re-irradiation with IMRT. Re-irradiation aggravated injuries caused by the first irradiation, which increased oxygen demand in these aggravated areas. At the same time, previous repair processes, such as fibrosis, may also constrain the local blood supply, which can hinder the healing of necrosis after re-irradiation. As previously reported, weekly debridement and excision of necrotic tissue under nasopharyngeal endoscopy, daily nasopharyngeal irrigation, intravenous nutrition, and systematic antibiotic therapy can improve some cases to a certain extent [15], which may diminish the need for procedures to treat nasopharyngeal necrosis. Thus, once nasopharyngeal necrosis is diagnosed, timely and effective treatment is necessary.

Finally, we found that women were more likely than men to develop LNN, suggesting that intrinsic biological traits, such as sensitivity to radiation, repair ability, and hormone levels, may contribute to a patient’s likelihood of developing LNN. Future investigations should study the relationship between necrosis and sex.

This study had several limitations. First, quick and effective measures are very important for hemorrhage rescue, but treatments of massive nasopharyngeal bleeding varied considering expense, distance, and clinical medical condition. Massive nasopharyngeal bleeding involving the internal maxillary artery could be rescued with effective treatment, such as hemostasis by gelatin sponge compression, nasopharyngeal packing, artery ligation, and other surgical measures [32]. However, carotid artery rupture is a common result of LNN, which results in a high mortality [15]. In this study, we could not analyze ruptured arteries because of insufficient clinical data. Second, this study had a relatively small patient population, which diminishes the relevance of the results. Last, as a retrospective study, the patient population’s clinical characteristics were diverse; therefore, an observational prospective study is necessary to validate this scoring system.

## Conclusions

For patients with NPC who underwent radical re-irradiation with IMRT, we devised a model, which includes sex, necrosis before re-irradiation, accumulated total prescription dose to the GTV, and recurrent tumor volume, which can effectively predict the risk of developing LNN. We expect that future investigations will suggest strategies to prevent or reverse the development of LNN.
